# Application of a low molecular weight antifungal protein from *Penicillium chrysogenum* (PAF) to treat pulmonary aspergillosis in mice

**DOI:** 10.1038/emi.2016.116

**Published:** 2016-11-09

**Authors:** Zoltán Palicz, Tamás Gáll, Éva Leiter, Sándor Kollár, Ilona Kovács, Kornél Miszti-Blasius, István Pócsi, László Csernoch, Péter Szentesi

**Affiliations:** 1 Department of Physiology, Faculty of Medicine, University of Debrecen, Debrecen H-4012, Hungary; 2 Department of Biotechnology and Microbiology, Faculty of Science and Technology, University of Debrecen, Debrecen H-4032, Hungary; 3 Department of Pathology, Kenézy Gyula Hospital, Debrecen H-4031, Hungary; 4 Department of Laboratory Medicine, Medical Faculty, University of Debrecen, Debrecen H-4012, Hungary

**Keywords:** antifungal protein, histology, mouse, pulmonary aspergillosis

## Abstract

PAF, a small antifungal protein from *Penicillium chrysogenum*, inhibits the growth of several pathogenic filamentous fungi, including members of the *Aspergillus* genus. PAF has been proven to have no toxic effects *in vivo* in mice by intranasal application. To test its efficacy against invasive pulmonary aspergillosis (IPA), experiments were carried out in mice suffering from IPA. Adult mice were immunosuppressed and then infected with *Aspergillus fumigatus.* After stable infection, the animals were inoculated with PAF intranasally at a concentration of 2.7 mg/kg twice per day. At this concentration—which is highly toxic *in vitro* to *A. fumigatus*—the mortality of the animals was slightly delayed but finally all animals died. Histological examinations revealed massive fungal infections in the lungs of both PAF-treated and untreated animal groups. Because intranasally administered PAF was unable to overcome IPA, modified and combined therapies were introduced. The intraperitoneal application of PAF in animals with IPA prolonged the survival of the animals only 1 day. Similar results were obtained with amphotericin B (AMB), with PAF and AMB being equally effective. Combined therapy with AMB and PAF—which are synergistic *in vitro*—was found to be more effective than either AMB or PAF treatment alone. As no toxic effects of PAF in mammals have been described thus far, and, moreover, there are so far no *A. fumigatus* strains with reported inherent or acquired PAF resistance, it is worth carrying out further studies to introduce PAF as a potential antifungal drug in human therapy.

## Introduction

The number of immunodeficient patients is rising as a consequence of modern therapies, and they are endangered by infections from a wide spectrum of microbes, including yeasts and filamentous fungi.^1^ Fungal infections are the most dangerous among them because their treatments are often ineffective owing to inherent or acquired antimycotic resistance of the fungi.^2^ Aspergillosis is one of the most challenging infections and is caused by various *Aspergillus* species, amongst which the most virulent and most wide-spread species is *Aspergillus fumigatus.*^3^ It can cause several diseases, including aspergilloma, allergic bronchopulmonary aspergillosis, and invasive aspergillosis.^4, 5^ In invasive pulmonary aspergillosis (IPA), hyphae spread in the lung tissue and cause coagulation necrosis, which makes round-shaped discrete nodules with hyphae inside, or fused lobular consolidations can be observed, consisting of acute inflammatory exudate in the alveoli with fungal proliferation.^5^ This process can destroy a lobe or the whole lung, which leads to high mortality.

The therapy for IPA includes surgical removal of the aspergilloma (fungal balls developed in cavities) and drugs, such as voriconazole, itraconazole, posaconazole, caspofungin, micafungin or amphotericin B (AMB).^6, 7^ Mortality remains high even with long-term combination therapy, and relapse is prevalent.^8^ Antifungal resistance can evolve through diverse mechanisms; probably the most important is the deregulation of antifungal resistance effector genes by point mutations in their transcriptional regulators or the genes coding for antifungal targets.^9^ In the last few years, more and more resistant *Aspergillus* strains have been reported (*A. fumigatus*^10^ and *A. terreus*^11^). For that very reason and the high mortality of the patients, it would be important to find new drugs against IPA.

Various eukaryotes, including fungi, produce a wide spectrum of small antifungal proteins, which are suitable for the development of new therapeutic agents against fungal diseases.^12, 13^ *Penicillium chrysogenum* produces not only penicillin but also a low molecular mass (6.5 kD) protein (PAF, GenBank accession number AAA92718).^14^ PAF is stable even under extreme test conditions (pH, high temperature and proteases)^15^ and possesses a fungicide effect on various species including some *Aspergillus* species, such as *A. fumigatus, A. niger* and *A. nidulans.*^16, 17^ The protein enters sensitive fungi through internalization via an endocytotic mechanism^18^ and elicits the increased production of harmful reactive oxygen species enhancing the oxidation of intracellular molecules, decreases the metabolism of fungal cells, and triggers disintegration of mitochondrial membranes.^16, 19, 20^

It was shown previously that PAF had no toxic effects on a wide range of mammalian cells.^21^ We demonstrated previously *in vivo* that intranasally applied PAF did not alter important physiological parameters of mice.^22^ At concentrations that are highly toxic for most sensitive molds *in vitro*, the animals neither died because of the treatment nor were there any side effects observed. It was confirmed that a detectable amount of PAF accumulated in the lungs, and it kept its antifungal activity for hours.

In this study, we tested PAF *in vivo* against pulmonary aspergillosis in mice. Single or combination therapy with amphotericin B—showing synergistic effects *in vitro*—prolonged the survival of animals suffering from IPA. Our results suggest that further animal investigations *in vivo* should be carried out to support the possible therapeutic application of PAF against aspergillosis in humans.

## Materials and Methods

### Purification of PAF

Purification of PAF was carried out as described previously.^21, 23^ Briefly, *P. chrysogenum* Q176 was grown in a sucrose (20 g/L) and NaNO_3_ (3 g/L) based minimal medium for 96 h at 25 °C with shaking. After harvesting of the mycelia by centrifugation and separation of the low molecular weight protein fraction in Amicon Stirred Cells (*V*=50 mL, Biomax PBTK ultrafiltration disks, size exclusion limit MWCO=30 000; MILLIPORE, Billerica, MA, USA), PAF was purified by ion-exchange chromatography on a CM Sephadex Fast Flow column (2 × 18 cm, equilibrated with 50 mm sodium phosphate buffer, pH=6.6, flow rate 1 mL/min, *t*=4 °C; AMERSHAM-Pharmacia, Uppsala, Sweden). PAF was eluted with a NaCl gradient (0.05–1 m) prepared in the equilibrating buffer, and PAF-containing fractions were dialyzed in phosphate-buffered saline (PBS).

### *In vitro* study assay

Minimum inhibitory concentration (MIC) determination of AMB and PAF and the drug combination studies were performed according to CLSI M38-A2 guidelines.^24^ The final concentrations of the tested drugs ranged from 0.0625 to 16 μg/mL for AMB, and from 25 to 500 μg/mL for PAF. The MICs of the antifungal agents were determined after 48 h of incubation at 35 °C as the lowest drug concentration at which there was complete inhibition of growth. In a checkerboard assay, combinations of the two drugs were determined according to the MIC values of the drugs used alone.^25^ The interaction of AMB and PAF was assessed by calculating the fractional inhibitory concentration index (FICI) with the following formula: FICI=((MIC A in combination)/MIC A)+((MIC B in combination)/MIC B). The interaction is synergic if the FICI is ⩽0.5; indifferent if the FICI is >0.5 and ⩽4; and antagonistic if the FICI is >4.^25^

### Preparation of fungal conidia

Inocula of *A. fumigatus* AF293 (clinical isolate from human lung tissue) were prepared by culturing the test organisms on minimal nitrate medium.^26^ Freshly grown (6 days) conidia were harvested in PBS-0.01% Tween 80 and counted in a Bürker chamber.

### Animal care

Eight to 12-week-old Balb/c mice of both sexes (weighting 17–25 g) were used in all *in vivo* experiments. The animal experiments conformed to the guidelines of the European Community (86/609/EEC). The experimental protocol was approved by the institutional Animal Care Committee of the University of Debrecen (11/2008/DE MÁB). The mice were housed in plastic cages with mesh covers and fed with pelleted mouse chow and water *ad libitum*. Room illumination was an automated cycle of 12 h light and 12 h dark, and room temperature was maintained within the range of 22 –25 °C.

Mice infected with *A. fumigatus* were kept in isolation cages with high-efficiency particulate air filters. These cages were continuously ventilated, and slightly negative air pressure was used to prevent infection from fungal spores. Each cage was inspected at least three times daily.

### Infection of mice with *A. fumigatus*

Mice were immunosuppressed with 250 mg/kg cyclophosphamide (Endoxan, Baxter, IL, USA) intraperitoneally (IP) three days before and one day after infection with *A. fumigatus* conidia. A total of 3.5 × 10^6^ conidia dissolved in 50 μL PBS was given to mice intranasally under anesthesia with isoflurane. The number of conidia was chosen to produce 100% mortality. This was tested in preliminary experiments (Supplementary Figure S1). Gentamicin was added to the water of the mice (5 mg/kg). The survival of the animals was monitored three times daily after infection. All infected animals were humanely terminated if they presented severely reduced mobility (i.e., unable to reach their water or food) or substantial distress. After the death of animals, their organs were removed and histological examination was carried out.

### Intranasal PAF treatment of animals suffering from invasive pulmonary aspergillosis

In five independent experiments, immunosuppressed mice were randomly divided into three groups from which two were *Aspergillus* conidia infected. Among the infected groups the first group was treated with 5.4 mg/kg PAF (26 mice), the second group with PAF-free PBS (positive control, 23 mice) intranasally in the final volume of 50 μL. The intranasal application of PAF was described earlier.^22^Briefly, mice were slightly anesthetized with isoflurane, then 2.7 mg/kg PAF dissolved in PBS were administered by pipette into the nose of the animals in a 50 μL final volume. Under these conditions the solution reaches the lungs through normal breathing. Treatments lasted until the death of animals from the day of the infection twice a day (morning and afternoon). The control group (negative control, six mice) was not infected but immunosuppressed and treated intranasally with PAF-free PBS twice a day. Some animals from the infected group were killed 3 days after the infection, and their organs were examined histologically.

### Intraperitoneal PAF treatment of healthy animals

Mice were randomly divided into two groups. The first group (*n*=8) was treated IP with 5.4 mg/kg PAF dissolved in 100 μL PBS twice a day and intranasally twice a day with 2.7 mg/kg PAF dissolved in 50 μL PBS for seven days. The second (control) group (*n*=8) was treated with PAF-free PBS in the same way. On the eighth day, some animals were killed and the organs were removed for histological examination. On the first and last days of the experiment, body weight was measured, blood was collected, and blood cell count and other plasma parameters were determined.^22^

### Combined treatment with PAF and amphotericin B of animals suffering from invasive pulmonary aspergillosis

In two independent experiments, immunosuppressed mice were randomly divided into five groups (all together 11 mice per group). Animals in four groups were *Aspergillus* conidia infected, the non-infected group was the negative control. All antifungal treatment started on the day of the fungal infection and lasted till the death of the animals. The first infected group was treated with PAF and amphotericin B-desoxycholate (Fungizone, Bristol-Myers Squibb, Montreal, QC, Canada), the second group with PAF, the third group with AMB and the positive control group only with PBS. A total of 5.4 mg/kg PAF dissolved in PBS was injected IP twice a day in 100 μL final volume, and 50 μL were administered by pipette into the nostrils of the animals under anesthesia once a day. One hundred microliters AMB were injected IP in 5 mg/kg concentration three times as follows: one, two and four days after the fungal infection.^27^ The animals in the negative control group were treated only with PBS in the same way as the PAF-treated group. After the death of the infected animals, their organs were removed for histological examination.

### Histological examination and lung injury score

To explore the possible positive effects of intranasal and intraperitoneal application of PAF, the lungs, the kidneys and the liver of the animals were examined histologically as described earlier.^22^ Briefly, tissue samples were fixed in 8% buffered formalin (24 h) and embedded in paraffin wax (Shandon Pathcenter, Thermo-Shandon, Astmoor, WA, USA). Hematoxylin–eosin (H&E) and periodic acid–Schiff (PAS) staining was performed on 4 μm thick cut sections. The alveolar exudate/edema and hemorrhage were scored on a scale of 0–3 (0, absent; 1, mild; 2, moderate; 3, severe) from five randomly chosen fields in each sample.

### Blood collection and cell counting

Blood samples were taken as described earlier.^22^ Briefly, 200 μL blood was collected at the beginning and at the end of the 1-week long experiment from control animals (*n*=8) and animals treated IP and intranasally with 10.8 mg/kg and 5.4 mg/kg doses of PAF (*n*=8), respectively. Anticoagulated whole blood was analyzed by a Siemens Advia-120 hematology analyzer (Deerfield, IL, USA). The numbers of white blood cells and neutrophil granulocytes, the levels of liver enzymes and ions in the plasma were determined to explore the possible toxic effects of PAF.

### Chemicals and statistical analysis

Chemicals, unless otherwise stated, were purchased from Sigma (St. Louis, USA) and were of analytical grade. Averages were expressed as the means±standard error (SE) of the mean. The differences between control and treated animals were assessed using one-way analysis of variance (ANOVA) and all pairwise multiple comparison procedures (Student–Newman–Keuls method). The F test was used to test significance, and a *P* value of <0.05 was considered statistically significant.

Percentage survival was computed with the Kaplan–Meier method. The mortality rate as a function of time was fitted to a Gompertz mortality rate equation.^28^ The equation is as follows:


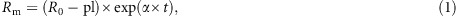


where *R*_0_ is the initial mortality rate, pl is plateau at the end of experiment, *α* is the Gompertz parameter and *t* is the time. The significance of differences between survival curves was assessed as the statistically accepted differences in the fitted Gompertz parameter (*α*) and with the log-rank (Mantel–Cox) test. The results were evaluated with GraphPad Prism 6.0 software (La Jolla, CA, USA).

## Results

### Survival and histological examination of animals suffering from pulmonary aspergillosis treated intranasally with PAF

**Figure 1:**
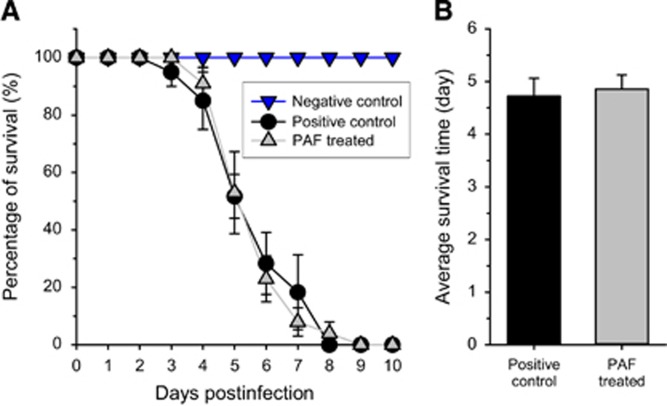
Survival of mice suffering from pulmonary aspergillosis with and without nasal PAF treatment. (**A**) Survival curves obtained from five independent experiments of untreated (black ●, *n*=23) and 5.4 mg/kg PAF-treated (gray ▲, *n*=26) mouse groups infected intranasally with 3.5 × 10^6^ *Aspergillus fumigatus* conidia/50 μL phosphate-buffered saline (PBS). The negative control (no fungal infection, blue ▼, *n*=6) shows 100% survival. (**B**) Average survival time of fungal infected mice without (positive control) and with PAF treatment (PAF). Error bars represent the standard error.

As the most likely application of PAF in human therapy is expected to be the treatment of lung aspergillosis, which implies the administration of the drug via the airways, we simulated this procedure by introducing PAF intranasally to mice at a high concentration (5.4 mg/kg). Infected animals suffering from IPA were evidently sick, moved slowly and their fur was matted. Moreover, their weight decreased significantly (Table 1). PAF delayed the death of animals by ~1 day, but all animals had died by day nine (Figure 1A). Log-rank analysis showed no significant differences (*P*=0.86) between the survival curves. In contrast with this, all of the control non-infected mice, treated only with PAF in PBS, showed 100% survival and their body weight was stable (Table 1). Overall, intranasal PAF treatment did not increase significantly (F_(1,47)_=0.23, *P*=0.63) the average survival of the animals (Figure 1B).

**Figure 2:**
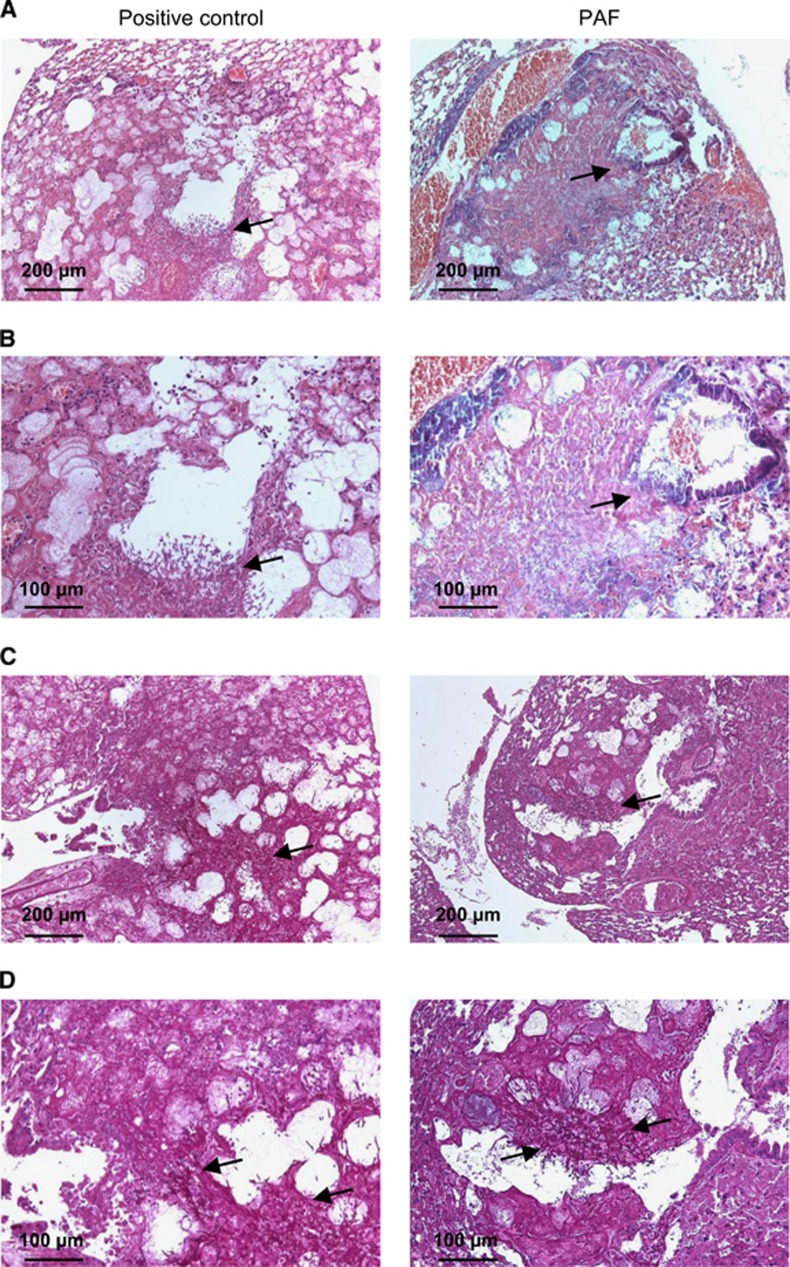
Histological investigation of lung tissue from mice suffering from pulmonary aspergillosis with and without nasal PAF treatment. Representative images show histological features of lung tissues stained with hematoxylin–eosin (H&E) (**A** and **B**) and PAS (**C** and **D**) from untreated positive control (left column) and 5.4 mg/kg PAF-treated (right column) mice suffering from invasive pulmonary aspergillosis (IPA) at two magnifications. Extensive fungal growth and tissue damage are evident in the untreated IPA mice. Less extensive fungal infection is present in the PAF-treated mice. Arrows show fungal balls with high density fungi and proliferating hyphae. See the strong magenta colored hyphae in **C** and **D**.

Histological examination of the lungs displayed severe pulmonary lesions characterized by multifocal to coalescent infiltrations of macrophages and neutrophils associated with vascular phenomena (thrombi, necrosis and hemorrhages) and alveolar and bronchiolar epithelial cell necrosis. There were no remarkably positive effects in the lungs following intranasal application of the antifungal protein. In positive control mice, a high number of proliferating hyphae (Figure 2, left panels) and coagulation necrosis were observed in the blood vessels, septa, bronchioles and alveoli. In the lungs from mice treated intranasally with PAF, hyphae were also frequently observed in the bronchioles and the alveoli. However, invasion of hyphae into blood vessels was not observed (Figure 2, right panels). The lung injury score was significantly higher in both cases (6±0 and 5.3±1.2 in positive control and PAF-treated mice, respectively) than in uninfected negative control animals (0.3±0.3; F_(2,6)_=18.5, *P*=0.003). The PAF-treated animals had values slightly but not significantly lower than the untreated ones (F_(1,4)_=0.31, *P*=0.61). It is important to mention, however, that we did not find fungal infection in the kidneys or in the liver (data not shown).

### Effects of intraperitoneal application of PAF on healthy animals

As the nasal application of PAF was not as successful as expected, the drug was administered IP as well. Control non-fungal-infected mice were treated IP with 0 and 5.4 mg/kg doses of PAF twice a day for 1 week. Blood samples were collected at the beginning and end of the experiment (Table 2).

Although the total white blood cell count decreased slightly (F_(1,14)_=35.5, *P*=3 × 10^−5^), the percentage of neutrophils remained unchanged (F_(1,14)_=0.71, *P*=0.41). Furthermore, the concentration of liver enzymes and ions in the blood did not show significant (0.01<F_(1,14)_<4.39, 0.08<*P*<0.93) elevation/alteration after PAF treatment. There was no significant change in the weight of the animals during the application of PAF. The average weight of the animals was 17±0.3 g at the beginning and 17.2±0.4 g at the end (F_(1,14)_=0.19, *P*=0.67). These results suggest that the intraperitoneal application of PAF neither induced any overall changes in the animals nor initiated an overall immune response, similarly to the nasal application.

### Combined treatment of animals suffering from pulmonary aspergillosis

**Figure 3:**
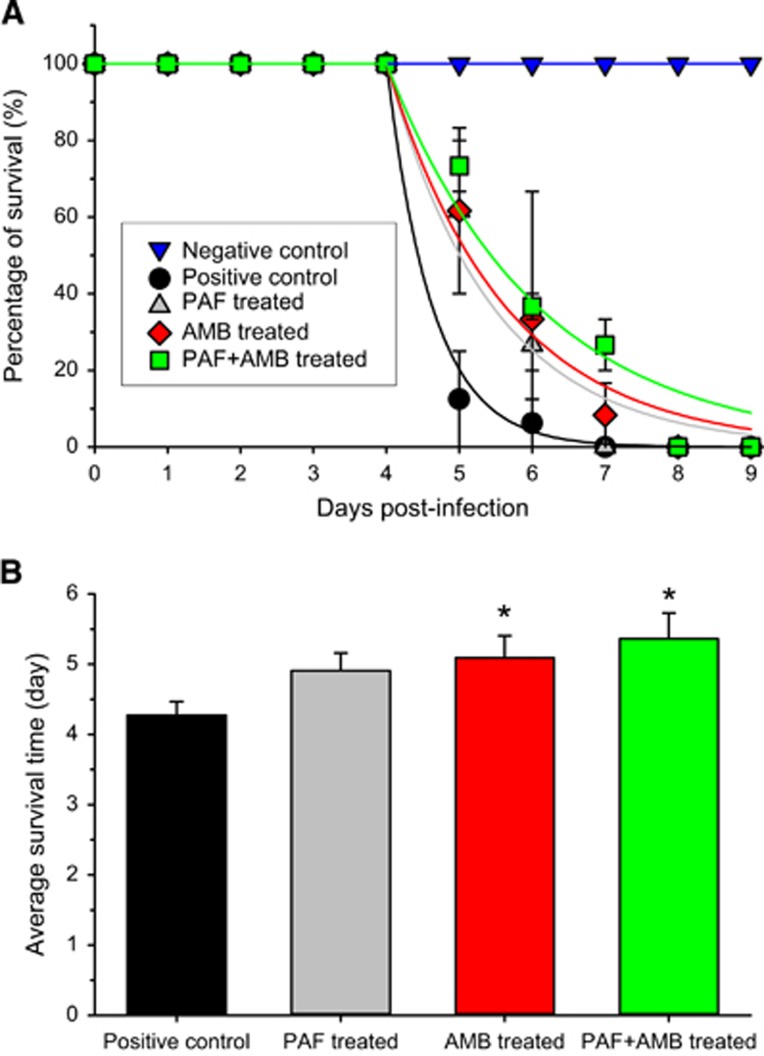
Survival of mice suffering from pulmonary aspergillosis with and without intraperitoneal PAF and amphotericin B (AMB) treatment. (**A**) Survival curves obtained from two independent experiments of untreated (black ●, *n*=11), PAF-treated (gray ▲, *n*=11), 5 mg/kg AMB-treated (red ♦, *n*=11) and combined PAF-AMB-treated (green ■, *n*=11) mouse groups infected intranasally with 3.5 × 10^6^ *A. fumigatus* conidia/50 μL PBS. The negative control (no fungal infection, blue ▼, *n*=11) shows 100% survival. Lines starting at day 4 in the infected groups represent the best fit of Equation (1) to the averages. (**B**) Average survival time of fungal infected mice without (positive control) and with PAF, AMB and combined (PAF+AMB) treatment. *Denotes significantly different from positive control (*P*=0.06, *P*=0.04 and *P*=0.02 for PAF, AMB and PAF+AMB treatment, respectively). Error bars represent the standard error.

To increase the positive effects of PAF, its intranasal application was complemented with intraperitoneal injection. The combined application of PAF and AMB was also tested. Before the *in vivo* application, an *in vitro* drug combination assay of AMB plus PAF was performed. With AMB and PAF alone, the MIC values against *A. fumigatus* AF293 proved to be 1 and 300 μg/mL, respectively. FICI values indicated an *in vitro* synergic effect when AMB was combined with PAF. The FICI value calculated from the individual and combined MICs (0.25 μg/mL of AMB and 50 μg/mL of PAF) of the drugs is 0.42. Figure 3A presents the survival of infected mice that were treated with PAF, amphotericin B (5 mg/kg), and both antifungal drugs concomitantly. It is clearly visible in Figure 3B that although neither PAF nor AMB alone increased survival, the combined application resulted in a remarkable extension of life expectancy. In this experiment, all infected animals died within 7 days independently of the treatment they received. In the untreated infected group, more than 80% of the animals died after 5 days. Importantly, on this day more than 50% of the PAF or AMB-treated animals were still alive. It is noteworthy that in the group receiving the combined treatment, only 30% of the animals died by the 5th day. After 6 days, all PAF-treated animals died, meanwhile 9% of the AMB-treated animals and 27% of the combined treatment animals were alive. Summing up, individual PAF or AMB treatment extended the survival of the animals by 1 day, and some animals in the combined treatment group lived 2 days longer than those in the untreated group.

To test the differences between the survival curves, Equation (1) was fitted to the data points (Figure 3A). Although the Gompertz mortality rate was high (*α*=1.596) in the positive control group, it was significantly lower in the PAF (*α*=0.6882, *P*<0.001) and AMB (*α*=0.6146, *P*<0.001) treated groups. Furthermore, the combined treatment significantly slowed down the mortality (*α*=0.4831, *P*<0.001) when compared with the untreated group. As all animals died in all groups, the plateaus of the curves were assumed to be zero. The log-rank test also proved that the survival curves of the treated groups are significantly different from that of the untreated one (*P*=0.04).

There was no significant difference in the average survival time (Figure 3B) between the single treatment groups (F_(1,20)_=0.20, *P*=0.66). PAF treatment alone caused a close to significant increase (F_(1,20)_=4.02, *P*=0.06) in the average survival of the animals. However, AMB alone (F_(1,20)_=4.88, *P*=0.04) and the combined PAF and AMB treatment (F_(1,20)_=6.99, *P*=0.02) increased significantly the lifetime of the animals.

**Figure 4:**
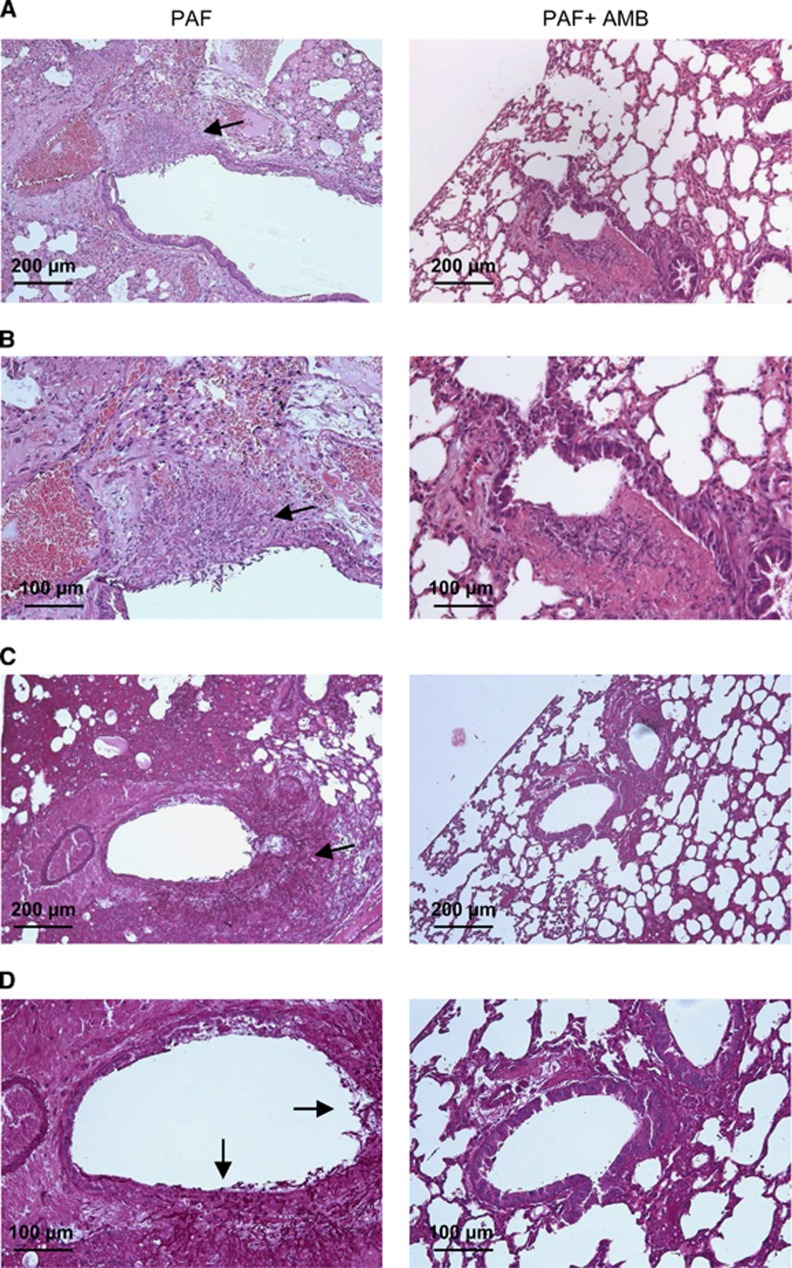
Histological investigation of lung tissue from mice suffering from pulmonary aspergillosis with intraperitoneal PAF and amphotericin B treatment. Representative images show histological features of lung tissues stained with H&E (**A** and **B**) and PAS (**C** and **D**) from PAF-treated (left column) and PAF plus amphotericin B (AMB)-treated (right column) mice suffering from invasive pulmonary aspergillosis (IPA) at two magnifications. Extensive fungal growth and tissue damage are evident in the PAF-treated IPA mice (arrows). Note the lack of fungal balls and hyphae in the lungs of animals with combined treatment.

These observations were validated by histological examination. In the lungs from IP PAF-treated mice, a number of proliferating hyphae (Figure 4, left panels) and coagulation necrosis were noted in bronchioles and alveoli. In the lungs from mice treated with PAF and AMB, hyphae were not observed (Figure 4, right panels). H&E staining of sections confirmed less extensive inflammation in the combined AMB and PAF-treated animals (Figures 4A and 4B). In addition, PAS staining proved the manifestation of extensive fungal infection in the single antimycotic (either PAF or AMB) treated animals and the lack of infection when PAF and AMB were used in combination (Figures 4C and 4D). Furthermore, the lung injury score was lower in the combined treatment group (4±0.6, F_(1,5)_=4.96, *P*=0.08) than in the control group (6.3±0.8). However, the score of the PAF-treated group (5±0.0) was almost identical to that of AMB-treated animals (5.3±0.6, F_(1,4)_=0.25, *P*=0.64). None of them was significantly lower than the control value (F_(2,7)_=1.11, *P*=0.38.)

### Combined treatment with a reduced concentration of AMB

**Figure 5:**
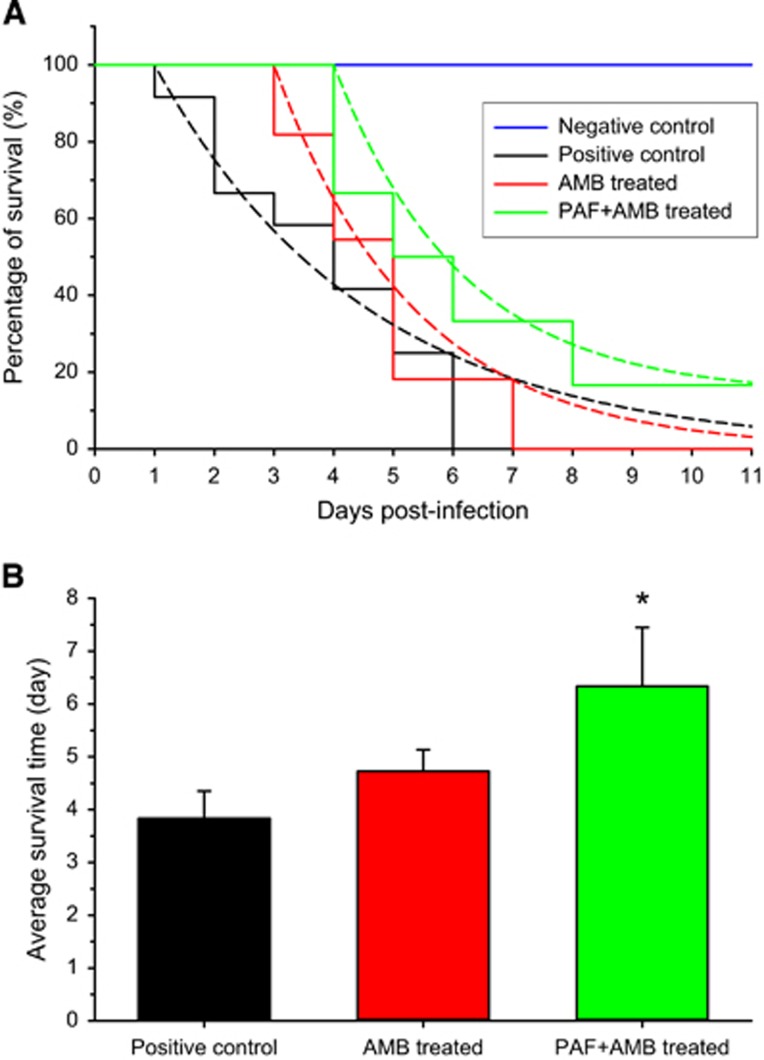
Survival of mice suffering from pulmonary aspergillosis treated with and without PAF and reduced dose of amphotericin B (AMB). (**A**) Survival curves obtained from one experiment of untreated (black, *n*=12), 2.5 mg/kg AMB-treated (red, *n*=11) and AMB combined with PAF (green, *n*=6) mouse groups infected intranasally with 3.5 × 10^6^ *A. fumigatus* conidia/50 μL PBS. The negative control (no fungal infection, blue, *n*=6) shows 100% survival. Dashed lines starting at days 1, 3 and 4 in infected groups represent the best fit of Equation (1) to the data. (**B**) Average survival time of fungal infected mice without (positive control) and with AMB or combined (PAF+AMB) treatment. *Denotes significantly different from positive control (*P*=0.20 and *P*=0.03 for AMB and PAF+AMB treatment, respectively). Error bars represent the standard error.

To investigate the efficacy of PAF, the previous experiment was repeated with a reduced concentration (2.5 mg/kg) of AMB. The combined application of PAF and AMB (*n*=6) was compared with the single AMB-treated group (*n*=11). Figure 5A shows that AMB alone delayed but did not prevent the death of the animals. After 8 days, all AMB-treated animals died, whereas its combined application with PAF not only delayed further the development of aspergillosis but also resulted in the survival of an infected mouse (16.6%). In contrast, all infected and untreated animals (*n*=12) died within seven days. The combined PAF and AMB treatment extended the survival of the animals by 1 day, and one animal recovered from aspergillosis.

Because the animals started to die earlier (at 2nd day) in the positive control group, the analysis of survival curves resulted in a slow (*α*=0.2822) Gompertz mortality rate. It was significantly higher in AMB (*α*=0.4292, *P*=0.002) treated groups, but the death of the animals started only at the 3rd day. In both cases the final number of animals (plateau) was 0. However, the combined treatment delayed the start of animal death to the 4th day, increased the survival of animals (plateau is 14.1%) and resulted in a significantly different mortality rate (*α*=0.4689, *P*=0.007) when compared with the untreated group. However, the log-rank test only showed significant difference for the trend (*P*=0.03) and not between the survival curves themselves (*P*=0.09).

There was no significant difference in the average survival time (Figure 5B) between the single treatment and positive control group (F_(1,21)_=1.79, *P*=0.20). However, the combined PAF and AMB treatment (F_(1,16)_=5.48, *P*=0.03) increased the lifetime of the animals significantly.

## Discussion

The present study compared the *in vivo* antifungal activity of PAF when administered either intranasally or IP alone or in combination with amphotericin B—as an *in vitro* interaction study confirmed that the effect of these drugs is synergistic against *A. fumigatus*—in an immunocompromised mouse model of invasive pulmonary aspergillosis initiated by *A. fumigatus*. The model was highly reproducible, and all untreated animals died within 9 days following the fungal infection.

Although PAF is a protein, it is highly stable,^15^ and, hence, it is easy and safe to prepare it for administration independent of the mode of application. In the case of nasal application, a concentration high enough to have antifungal activity *in vitro* was determined in the lungs.^22^ However, independent of the mode of application, the protein was undetectable in serum (data not shown). Notably, accumulation of PAF over time was not observed, which was most likely the consequence of its short half-life within the body. It should be noted that blood samples were taken 120 min after the last intraperitoneal administration of the drug and that PAF is small enough to be filtered by the glomeruli and thus excreted by the kidneys. On the basis of this, to achieve detectable concentrations of PAF in the serum of mice, the protein should be administered in doses higher than those used in our experiments. There may be a threshold therapeutic concentration for the successful medication of IPA, which should be determined in future studies.

The intraperitoneal administration of PAF was beginning to prevent the spread of fungi in the lungs around days 5 and 6 post-infection, but finally it was not able to overcome the fungal invasion. It is remarkable that PAF alone was almost as effective as the lower (2.5 mg/kg) concentration AMB, an antimycotic used frequently in the medication of IPA.^5, 29^ Furthermore, the combined application of PAF and AMB was clearly superior to either single antimycotic (PAF or AMB) treatment when increased survival times were considered. The dose of AMB (5 mg/kg) used in this model was not able to prevent the progression of IPA in mice. In human treatment, AMB is usually administered in intravenous (IV) infusion for 1–2 weeks, and its typical dosage is 3 mg/kg·day. With higher dosage of AMB (10 mg/kg·day), the incidence of nephrotoxicity increases.^30^ However, in patients with IPA, no difference was observed in efficacy between the low and high dosage of AMB showing ~50% positive effect. A comparative study with posaconazole and AMB found that although the survival with posaconazole was 70%–90%, it was only 0–50% with AMB.^31^ The efficiency of AMB and anidulafungin was also compared, and both antimycotics could prolong survival, but finally at least 90% of the animals died in every group.^32^ These findings are in good accordance with our observations.

We have to note notwithstanding that the intraperitoneal administration of AMB is less clinically relevant, but its synergic effects with PAF could also be investigated in our animal model. It was shown previously that the distribution of AMB in the lungs is significantly different when administered IP versus intravenously.^33^ As IP administration resulted in a higher level of AMB in the lungs than the IV route, in further animal studies the applied concentration should be different than was used here. It is also possible that the histological changes we observed were partly the consequence of the accumulation of AMB, although we used AMB at a low concentration and only three times.

It is also promising that the combined application of PAF with lowered AMB (2.5 mg/kg) was as effective or even better than the combined treatment with the high concentration AMB. This finding raises the possibility of combined treatment with reduced AMB and thus decreased toxicity, which has been observed in mice suffering from IPA.^34^ In contrast, no toxicities or side effects of PAF have been reported thus far.^22^

Similar to other pathogenic fungi, *Aspergillus* spp. can develop resistance against different antifungal agents. For example, itraconazole resistance was first reported in 1997,^35^ and more than 50% of the strains resistant to itraconazole were cross-resistant to other azoles, such as voriconazole and posaconazole, which are primary drugs in antifungal therapies.^10^ Resistance to antimycotics can emerge through different mechanisms, such as increased drug reflux, modification or over-expression of target enzymes, up-regulation of homeostatic stress-response pathways, exogenous cholesterol import or altered drug uptake.

Owing to the emergence and spread of resistance towards frequently used antifungals, there is an urgent need for new types of antimycotics effective against human opportunistic *Aspergillus* spp. Our study demonstrated for the first time that PAF has potential *in vivo* against IPA even when administered at relatively low concentrations. Furthermore, the fact that AMB interacted synergistically with PAF *in vitro* indicated that such combinations may also have good efficacy in the treatment of neutropenic patients suffering from IPA. Future *in vivo* studies should aim at shedding light on further interactions of antimycotic drugs with PAF and determining the threshold concentrations at which IPA could be blocked or even reversed.

## Figures and Tables

**Table 1:** Average body weight of mice in different treatments

	**Relative time to infection**		**F statistics**
	**−1 day**	**+3 days**	
Intranasal treatment			
Negative control (*n*=6)	17.9±0.7	17.2±0.4	F_1,10_=0.13, *P*=0.73
Positive control (*n*=23)	17.1±0.4	13.8±0.3^a^	F_1,44_=42.9, *P*=2 × 10^−4^
PAF-treated (*n*=26)	17±0.4	13.7±0.4^a^	F_1,50_=41.1, *P*=2 × 10^−4^
			
Intranasal and IP treatment			
Negative control (*n*=11)	18.5±0.2	17.2±0.7	F_1,20_=4.24, *P*=0.06
Positive control (*n*=11)	18.5±0.1	11.9±0.1^a^	F_1,20_=338, *P*=2 × 10^−8^
PAF-treated (*n*=11)	17.5±0.5	12.2±0.3^a^	F_1,20_=73.6, *P*=6 × 10^−5^
AMB-treated (*n*=11)	18.6±0.2	12.6±0.3^a^	F_1,20_=192, *P*=7 × 10^−8^
PAF+AMB-treated (*n*=11)	19.4±0.5	12.7±0.3^a^	F_1,20_=115, *P*=5 × 10^−6^

Abbreviations: Amphotericin B, AMB; intraperitoneally, IP.

a Denotes significantly different from body weight before fungal infection (−1 day).

Values are given in grams. Animals in the negative control groups were immunosuppressed but not infected by *A. fumigatus.*

**Table 2:** Important parameters of plasma from control and IP PAF-treated mice

	**Control**	**PAF**	**F statistics**
White blood cells (G/L)	9.75±0.51	5.87±0.41^a^	F_1,14_=35.53, *P*=3 × 10^−5^
Neutrophils (%)	20.1±2.6	23.2±2.5	F_1,14_=0.71, *P*=0.41
Creatinine (μmol/L)	14.1±2.1	13±1.5	F_1,14_=0.20, *P*=0.66
Total bilirubin (mmol/L)	1.68±0.11	1.63±0.11	F_1,14_=0.17, *P*=0.70
AST-GOT (U/L)	389.5±45.2	238.8±56	F_1,14_=4.39, *P*=0.08
ALT-GPT (U/L)	208.6±63.7	82.3±39.4	F_1,14_=1.73, *P*=0.22
Albumin (g/L)	35.4±0.5	37.3±1.3	F_1,14_=1.83, *P*=0.20
Ca (mmol/L)	2.41±0.01	2.49±0.07	F_1,14_=1.23, *P*=0.31
K (mmol/L)	7.73±0.38	7.56±0.41	F_1,14_=0.08, *P*=0.79
Na (mmol/L)	149.4±0.9	149.5±0.6	F_1,14_=0.01, *P*=0.93
Cl (mmol/L)	109.3±0.7	108.3±0.6	F_1,14_=1.04, *P*=0.33

Abbreviations: Aspartate aminotransferase, AST; alanine transaminase, ALT; glutamate oxaloacetate transaminase, GOT; glutamate pyruvate transaminase, GPT; intraperitoneally, IP.

a None of the values, except white blood cell count, following PAF treatment were significantly different from those in the control.
