# Supplementary material for: Application of a low molecular weight antifungal protein from Penicillium chrysogenum (PAF) to treat pulmonary aspergillosis in mice
Source: Emerg Microbes Infect. 2016 Nov 9;5(11):e114–. doi: 10.1038/emi.2016.116 (PMC5148020; doi:10.1038/emi.2016.116)
Supplement: Supplementary Figure 1 [file emi2016116x1.pdf]

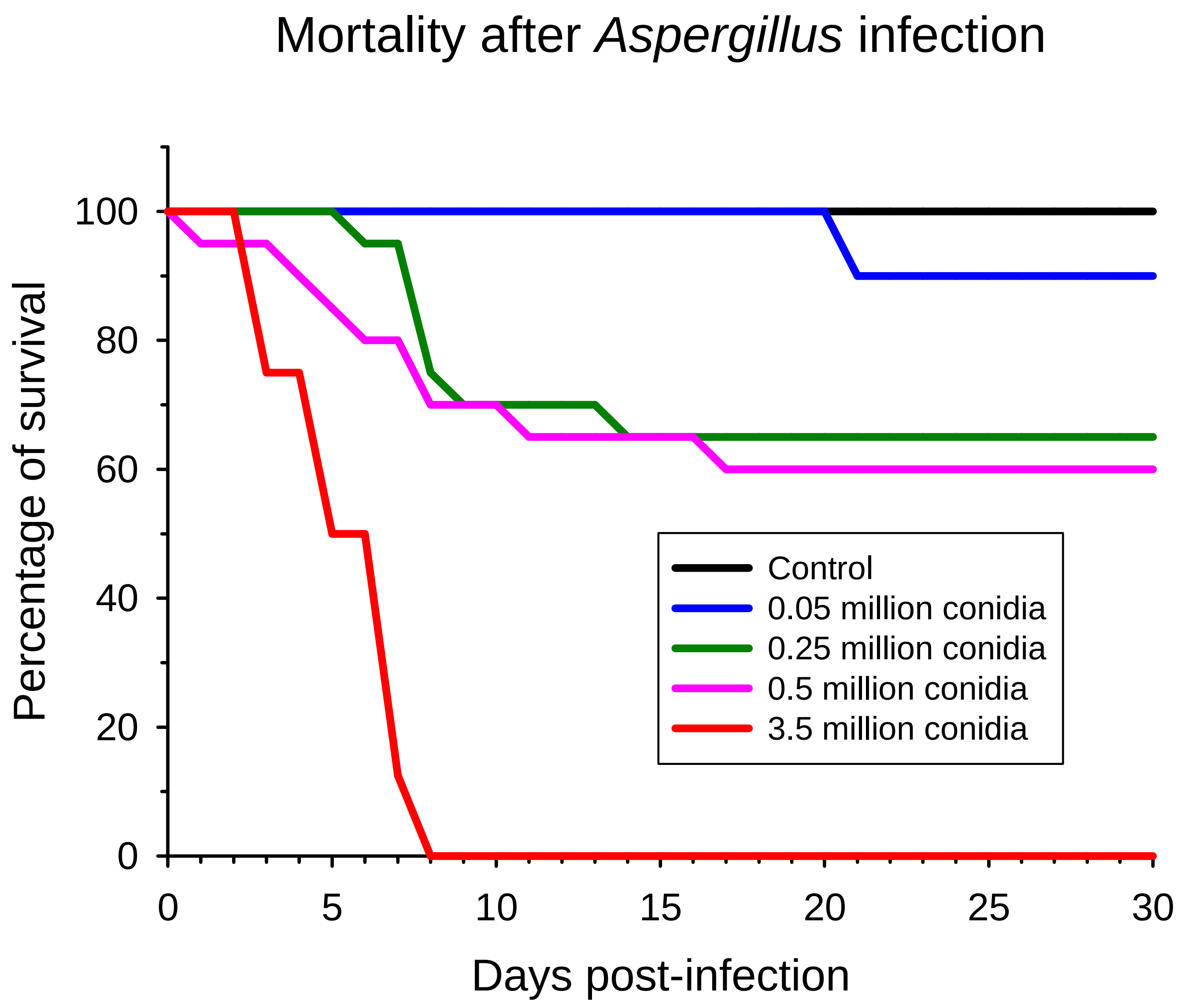

Supplementary figure 1. The dependence of mortality on the number of Aspergillus conidia

Immunosupressed mice were randomly divided into five groups (20 mice per group). Animals in four groups were *Aspergillus* conidia infected, the non-infected group was the control. The number of conidia was $5 \bullet 10^4$, $2.5 \bullet 10^5$, $5 \bullet 10^5$, and $3.5 \bullet 10^6$. The survival of the animals were monitored 30 days after infection.
